# Public Knowledge and Awareness About Hemorrhoids and the Reasons for Late Presentation to Local Doctors in Makkah Region, Saudi Arabia: A Cross-Sectional Exploratory Study

**DOI:** 10.7759/cureus.70349

**Published:** 2024-09-27

**Authors:** Mohamed E Salih, Abdullah S Alshumrani, Abdu Alrhman A Alodhayqi, Majed A Almdawsi, Ali K Almarhabi, Hussein A Alkhairi, Talal Q Alomary, Saud A Alshuqayfi

**Affiliations:** 1 Surgery, Umm Al-Qura University, Al Qunfudhah, SAU; 2 General and Colorectal Surgery, Wad-Medani Hospital, Medani, SDN; 3 Al-Qunfudah College of Medicine, Umm Al-Qura University, Al Qunfudhah, SAU

**Keywords:** hemorrhoids, knowledge, medical care, piles, population, prevalence, saudi arabia

## Abstract

Background

Hemorrhoids, also known as piles, manifest as anal lumps with or without bleeding. This study aims to explore and assess the public knowledge and awareness about hemorrhoids and the factors leading to late presentation to local doctors in the Makkah region of Saudi Arabia.

Methodology

A questionnaire-based, cross-sectional study was conducted targeting the Saudi population aged 18 years and more living in the Makkah region. The study was conducted from May 1 to May 29, 2023. Data collection was done using electronic Google Forms for data collection. The questionnaire was divided into three main sections covering demographics, knowledge items, and personal and family history of hemorrhoids.

Results

A total of 495 participants completed the study questionnaire. Participants’ ages ranged from 18 to 65 years with a mean age of 25.1 ± 13.7 years old. Overall, 414 (83.6%) were males. Only 106 (21.4%) had an overall good knowledge level. The majority of the participants (78.6%, 389) had poor knowledge. Pain (78% of the participants) was the most common reason for seeking medical advice, followed by feeling uncomfortable (43.3%), and the presence of blood or blood spots in the stool (41.1%). Among others who never sought medical advice, the most reported reason was being embarrassed (66.7%).

Conclusions

This study revealed that the prevalence of early seeking medical advice was low as many did not because of stigma. The main reasons for seeking medical care were disease-related symptoms, and embarrassment was the main reported barrier.

## Introduction

The frequency of anorectal diseases in the general population is 5-15% [1,2]. The incidence of anorectal diseases during pregnancy may increase up to 44%. The most frequent anorectal disorders are hemorrhoids and anal fissures [3-5]. When hemorrhoids, also known as piles, develop inside the rectum, they are called internal hemorrhoids, and when they develop at the anal verge, they are called external hemorrhoids [6,7]. Three-quarters of adults develop hemorrhoidal symptoms from time to time. Although the exact cause of hemorrhoids is not known, there are several risk factors [8,9]. Although it is not clear how hemorrhoids enlarge, some factors contribute to their development such as straining because of chronic constipation [10].

The most common classification system for hemorrhoids is Goligher’s classification which is based on the degree of prolapse. In Grade 1, there is bleeding, but no prolapse. Grade 1 hemorrhoids are discovered only with a proctoscope. Grade 2 piles prolapse when strained but subside spontaneously. Grade 3 piles prolapse but are manually reduced. Grade 4 are irreversibly prolapsing piles [11].

The exact prevalence of hemorrhoids has not been evaluated because patients usually do not present early and the estimated incidence worldwide is variable [11]. A study from Saudi Arabia showed that 66% of patients visit the hospital more than one year after the onset of symptoms [12]. Another study by Zaini et al. [13] in 2019 focused on the evaluation of anemic hemorrhoids. In summary, the different studies showed an awareness gap regarding hemorrhoid risk factors, possible complications, and the importance of seeking medical advice earlier. This study aimed to assess public knowledge regarding hemorrhoids in the Makkah region of Saudi Arabia.

## Materials and methods

In this cross-sectional study, we used an electronic questionnaire targeting all adults aged 18 years and above in Makkah. The study was done from May 1 to May 29, 2023. Individuals younger than 18 years were excluded together along with those who had not completely answered all the questions. Data were collected from the participants who fulfilled the research conditions using electronic Google Forms distributed through social media platforms. We first validated the questionnaire using a pilot study of 15 participants to assess the clarity, validity, and reliability of the questions concerning the study objectives. All questionnaire items were reviewed in the context of the study objectives to determine their content validity for all questionnaire domains (knowledge domain). The assessment was first done independently, and then items with arguments were discussed in detail to reach a consensus. All suggested changes were applied to improve the validity of the questionnaire. A pilot of 15 participants was used to assess questionnaire reliability with an alpha Cronbach’s value of 0.71. A total study sample of 370 participants was required to assess an average public awareness level about hemorrhoids at 69.7% with a precision of 5% at a 95% confidence level. The sample size was calculated using Epi-Info 7 software based on previously reported parameters.

The questionnaire was divided into three main sections. The first section included questions on demographics. The second section included closed questions to explore the participants’ knowledge and awareness about hemorrhoids. The third section included closed questions to explore the personal and family history of hemorrhoids, medical consultation, and reasons for visiting/not visiting doctors. The eligible respondents were asked to fill in the study questionnaire.

The questionnaire was sent to 600 participants, of whom 495 completed the questionnaire with a response rate of 82.5%. The entire research team was involved in the questionnaire distribution and data collection. Training and calibration were done by the entire research team meeting together and agreeing to a standard of distribution of the questionnaire and an agreed way of data collection.

Ethical approval was obtained from the Biomedical Ethics Committee of Umm Al-Qura University (approval number: HAPO-02-K-012-2023-04-1588). Electronic consent of the participants was acquired after they were made aware of the objectives of the study. They were informed that the data collected would be used for scientific research.

Data analysis

The data collection, revision, and analysis were performed using SPSS version 21 (IBM Corp., Armonk, NY, USA). All statistical methods used were two-tailed with an alpha level of 0.05 considered significant if the p-value was less than or equal to 0.05. Regarding knowledge and awareness, each correct answer was given a one-point score. Overall awareness level regarding hemorrhoids was evaluated by summing up discrete scores for different correct awareness items. The overall awareness score was considered poor if the participants’ score was less than 60% of the overall score and good if the participants’ score was 60% or more of the overall score. Descriptive analysis was done by proposing frequency distribution and percentage of study variables, including participants’ personal data, education, and personal and family history of hemorrhoids. Further, knowledge and awareness items for hemorrhoids were tabulated while overall knowledge level and reasons for visiting/not visiting doctors were graphed. Cross-tabulation showing the distribution of participants’ overall knowledge level by their data was performed using Pearson’s chi-square test for significance and exact probability test if there were small frequency distributions.

## Results

A total of 495 participants completed the study questionnaire. Participants’ ages ranged from 18 to 65 years with a mean age of 25.1 ± 13.7 years. Overall, 414 (83.6%) were males, 298 (60.2%) were single, and 191 (38.6%) were married. Regarding education, 275 (55.6%) were university graduates, 171 (34.5%) had a secondary level of education, and 20 (4%) had a postgraduate degree (Table 1).

**Table 1:** Personal characteristics of study participants in Makkah, Saudi Arabia.

Personal data	Number	%
Age in years		
18–25	259	52.3%
26–33	93	18.8%
34–41	42	8.5%
42–49	52	10.5%
50–57	36	7.3%
58–65	13	2.6%
Gender		
Male	414	83.6%
Female	81	16.4%
Marital status		
Single	298	60.2%
Married	191	38.6%
Divorced/Widow	6	1.2%
Educational level		
Below secondary	29	5.9%
Secondary	171	34.5%
University	275	55.6%
Postgraduate	20	4.0%

Table 2 shows participants’ knowledge and awareness regarding hemorrhoids in Makkah. Overall, 253 (51.1%) participants reported that hemorrhoid is a disease caused by swelling of the veins in the anal canal. Regarding causes of hemorrhoids, the most reported included sitting for long periods on the toilet (59.8%), having chronic diarrhea or constipation (51.5%), straining to move bowel (38.4%), and having a family history of hemorrhoids (25.9%). Pregnancy as one of the causes was reported by 13.3%.

**Table 2:** Participants knowledge and awareness regarding hemorrhoids in Makkah, Saudi Arabia

Knowledge items	Number	%
What are hemorrhoids?		
A disease caused by swelling of the veins in the rectal canal	253	51.1%
A cut in the tissue of the anus and rectum	119	24.0%
A disease that is transmitted through sexual intercourse	24	4.8%
A malignant tumor due to distension and swelling of the veins in the rectal canal	6	1.2%
Don’t know	93	18.8%
Causes of hemorrhoids		
Straining during a bowel movement	190	38.4%
Sitting for long periods on the toilet	296	59.8%
Having chronic diarrhea or constipation	255	51.5%
Eating a low-fiber diet	156	31.5%
Drink plenty of fluids	15	3.0%
Eating high-fiber foods	23	4.6%
Going to urinate as soon as you feel the urge	20	4.0%
Sport exercise	4	0.8%
Smoking	55	11.1%
Walking long distances	18	3.6%
Obesity	92	18.6%
Over the age of 50 years	4	0.8%
Pregnancy	66	13.3%
Having a family history of hemorrhoids	128	25.9%
Spicy food	11	2.2%
Based on what hemorrhoids are classified?		
Based on their external appearance	68	13.7%
Based on the extent of pain for the patient	142	28.7%
Based on the location of the dentate line in the anus	165	33.3%
Don’t know	120	24.2%

**Figure 1:**
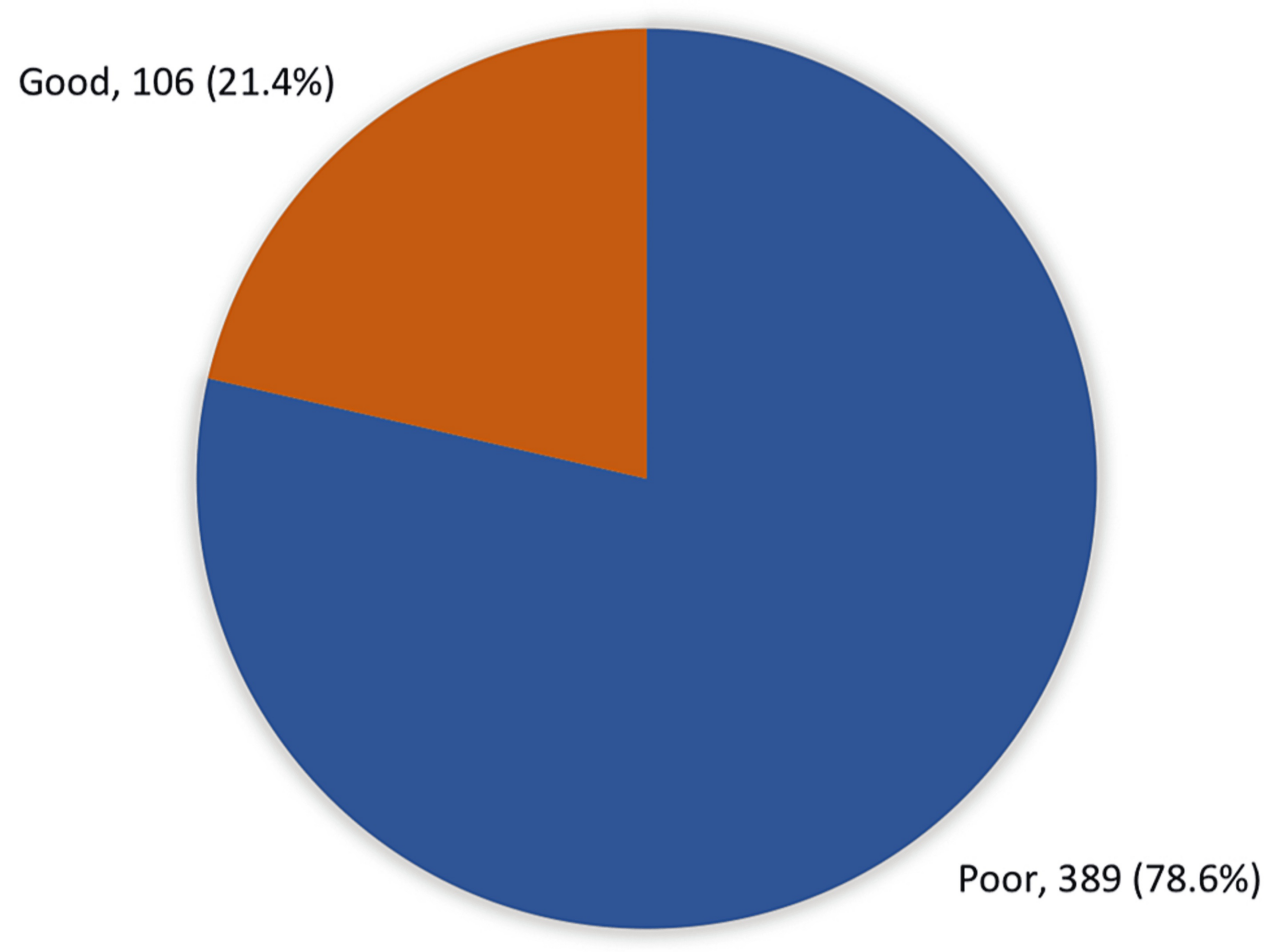
Overall knowledge and awareness regarding hemorrhoids in Makkah, Saudi Arabia.

Figure 1 shows the overall knowledge and awareness regarding hemorrhoids in Makkah. Only 106 (21.4%) had an overall good knowledge level regarding hemorrhoids while most of the participants (78.6%; 389) had a poor knowledge level.

As shown in Table 3, 49 (9.9%) had hemorrhoids, and 92 (18.6%) had a family history of hemorrhoids. In total, 24 (17%) participants had undergone hemorrhoid surgery, and 70 (49.6%) had relatives who had undergone surgery. Overall, 80 (56.7%) of those who complained of hemorrhoids visited their doctors in less than one year, 26 (18.4%) within one to two years, and 31 (22%) sought medical advice after three years.

**Table 3:** Personal and family history of hemorrhoids among study participants.

Hemorrhoid history	Number	%
Have you/any of your relatives had hemorrhoids?		
My self	49	9.9%
My relative	92	18.6%
None	354	71.5%
Have you/any of your relatives had hemorrhoid surgery?		
Myself	24	17.0%
My relative	70	49.6%
None	47	33.3%
If you had hemorrhoids before, how long did it take you to go to the doctor?		
<1 year	80	56.7%
1–2 years	26	18.4%
2–3 years	4	2.8%
>3 years	31	22.0%

**Figure 2:**
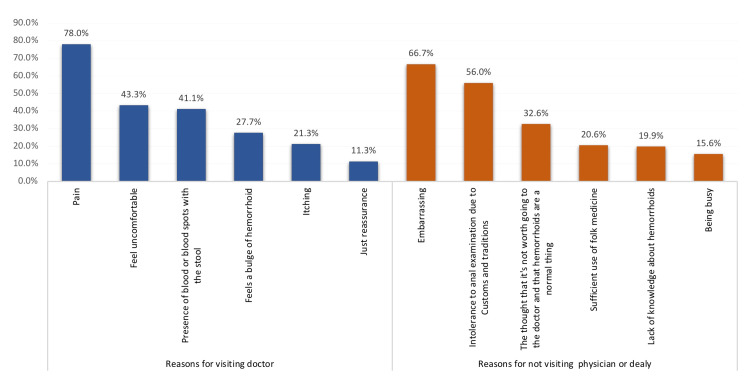
Reasons for visiting/not visiting doctors or delay among study cases diagnosed with hemorrhoids.

Figure 2 shows that most of the participants reported pain (78%) as a reason to visit the local doctor, followed by feeling uncomfortable (43.3%), the presence of blood or blood spots in the stool (41.1%), and feeling a bulge of hemorrhoid (27.7%). The main reason for not seeking medical advice was being embarrassed (66.7%), intolerance to anal examination due to traditions (56%), thinking that it is not worth going to the doctor and that hemorrhoids are not a disease (32.6%), sufficient use of folk medicine (20.6%), lack of knowledge about hemorrhoids (19.9%), and being busy (15.6%).

As shown in Table 4, 28.4% of female participants had good knowledge about hemorrhoids compared to 20% of males with recorded statistical significance (p = 0.049). Moreover, 40% of highly educated participants had a good knowledge level compared to 10.3% of others with a low educational level (p = 0.036). All other factors showed insignificant relation with participants’ knowledge level.

**Table 4:** Factors associated with participants’ knowledge about hemorrhoids in Makkah, Saudi Arabia. P: Pearson’s chi-square test; $: exact probability test; *: p < 0.05 (significant).

Factors	Overall knowledge level				P-value
	Poor		Good		
	No	%	No	%	
Age in years					0.588
18–25	202	78.0%	57	22.0%	
26–33	71	76.3%	22	23.7%	
34–41	35	83.3%	7	16.7%	
42–49	43	82.7%	9	17.3%	
50–57	26	72.2%	10	27.8%	
58–65	12	92.3%	1	7.7%	
Gender					0.049*
Male	331	80.0%	83	20.0%	
Female	58	71.6%	23	28.4%	
Marital status					0.762^$^
Single	231	77.5%	67	22.5%	
Married	153	80.1%	38	19.9%	
Divorced/Widow	5	83.3%	1	16.7%	
Educational level					0.036*
Below secondary	26	89.7%	3	10.3%	
Secondary	141	82.5%	30	17.5%	
University	210	76.4%	65	23.6%	
Postgraduate	12	60.0%	8	40.0%	
Have you/any of your relatives had hemorrhoids?					0.167
My self	34	69.4%	15	30.6%	
My relative	70	76.1%	22	23.9%	
None	285	80.5%	69	19.5%	
If you had hemorrhoids before, how long did it take you to go to the doctor?					0.091^$^
<1 year	64	80.0%	16	20.0%	
1–2 years	17	65.4%	9	34.6%	
2–3 years	4	100.0%	0	0.0%	
>3 years	19	61.3%	12	38.7%	

## Discussion

Hemorrhoids are swollen veins in the lower rectum or anus. They can be internal or external and can be a source of bleeding, itching, or discomfort. Risk factors leading to the formation of hemorrhoids are straining to empty the bowel, lengthy periods of sitting, and pregnancy [14]. Treatments include operative and non-operative such as creams and ointments, sitz baths, and lifestyle changes such as increasing fiber intake and staying hydrated [15]. In late or complicated cases, surgery may be necessary. It is advisable for anyone with symptoms of hemorrhoids to seek medical advice.

This study aimed to explore and assess the level of awareness, knowledge, and causes of hemorrhoids and assess the reasons for late medical consultation. Overall, 9.9% of the participants had hemorrhoids. Most of the participants visited their doctors within the first year of diagnosis mainly due to pain, discomfort, and having blood in the stool. Those who did not seek medical care were mainly embarrassed by being tagged with hemorrhoids or undergoing per rectal examination. Hemorrhoids are thought to affect 4.4% of the general population globally. The global prevalence of hemorrhoids is higher in Australia (38.93%) and Korea (14.4%). Australia (38.93%) has a greater hemorrhoid prevalence compared to Korea (14.4%). Very few trials have been conducted to evaluate the prevalence of hemorrhoids in Africa. The incidence of hemorrhoids among Egyptians undergoing colonoscopies was 18% [16]. Everhart and Ruhl [17] in the United States documented that nearly 3.3 million ambulatory care visits were due to hemorrhoid-related complaints, which makes it the fourth most common gastrointestinal disease in terms of outpatient diagnosis. Riss et al. [18] in Austria showed that the prevalence was 38.9%, and most of them were asymptomatic. Ismail et al. [19] in Somalia conducted a study among the general population and revealed that participants with higher socioeconomic status had higher incidence rates for hemorrhoids. Another survey by Sheikh et al. [20] which included multiple countries revealed that females had a higher prevalence than males, accounting for 52% of all hospital admissions.

This study showed that nearly one-fifth of the participants had a good knowledge level regarding the disease. About half of the participants reported that hemorrhoid is a disease caused by swelling of the veins in the rectal canal. Sitting for long periods on the toilet was considered by the majority as the main cause of hemorrhoids, with other causes including chronic diarrhea or constipation, straining to move the bowel, and having a family history of hemorrhoids. Female gender with high education was significantly associated with a high knowledge level. In a study by Rayzah [21] in Majmaah city regarding hemorrhoids, a low-fiber diet accounted for 89.8% of cases as the cause of hemorrhoids, while 71.6% reported family and friends as their source of knowledge about hemorrhoids. Another study among medical students and the general population revealed that 2.5% of the participants had never heard about hemorrhoids. Overall, about half of the participants stated straining to move the bowel as a cause of hemorrhoids. In addition, about 28.7% reported diarrhea as a precipitant cause of piles. Lifestyle was one factor that was reported in 45.9% of responses [22]. Alamri et al. [23] found that 46.4% of participants thought constipation was a cause of hemorrhoids, while 52.8% selected prolonged sitting in the toilet as a cause. Paszko et al. [24] documented that most of the participants stated straining, constipation, and sedentary lifestyle as causes of hemorrhoids, while only 50% of them thought low fiber intake to be a cause.

Limitations

This study had a few limitations. The results cannot be generalized because of the small research population, limited sociocultural group, and strong local beliefs, especially those related to men bleeding from the lower part of the gastrointestinal system. The local community in the researched area strongly adheres to traditional beliefs despite the local media and efforts by local health authorities to educate and encourage them to early report any health-related issues to the local health authorities. The time frame for the entire research was short which was meant to meet the deadline for submission to the medical college research authorities. Large research samples from different regions of the country are needed over a reasonably lengthy research period with more research staff to generalize the results.

## Conclusions

This study revealed that the prevalence of early self-reported hemorrhoids was low because of embarrassment, being asymptomatic, or not knowing about the condition. The main reasons for seeking medical care were disease-related symptoms and, on the other hand, embarrassment was the main reason for late presentation to local doctors. In addition, participants’ knowledge regarding hemorrhoids was poor. Greater knowledge was noticed among female participants with high education. We think more efforts are necessary to expand public awareness through a variety of strategies, including health education sessions, mass media, and healthcare staff in healthcare centers. Likewise, improving lifestyles will help in the prevention of disease-associated complications.
